# Ethnic differentials in under-five mortality in Nigeria

**DOI:** 10.1080/13557858.2014.890599

**Published:** 2014-03-05

**Authors:** Sunday A. Adedini, Clifford Odimegwu, Eunice N.S. Imasiku, Dorothy N. Ononokpono

**Affiliations:** ^a^ Programme in Demography and Population Studies, University of the Witwatersrand, Johannesburg, South Africa; ^b^ Department of Demography and Social Statistics, Obafemi Awolowo University, Ile-Ife, Nigeria; ^c^ Department of Geography, University of Zambia, Lusaka, Zambia; ^d^ Department of Sociology and Anthropology, University of Uyo, Uyo, Nigeria

**Keywords:** inequality, under-five mortality, ethnicity, cultural practices, Nigeria

## Abstract

**Objective.** There are huge regional disparities in under-five mortality in Nigeria. While a region within the country has as high as 222 under-five deaths per 1000 live births, the rate is as low as 89 per 1000 live births in another region. Nigeria is culturally diverse as there are more than 250 identifiable ethnic groups in the country; and various ethnic groups have different sociocultural values and practices which could influence child health outcome. Thus, the main objective of this study was to examine the ethnic differentials in under-five mortality in Nigeria.

**Design.** The study utilized 2008 Nigeria Demographic and Health Survey (NDHS) data. We analyzed data from a nationally representative sample drawn from 33,385 women aged 15–49 that had a total of 104,808 live births within 1993–2008. In order to examine ethnic differentials in under-five mortality over a sufficiently long period of time, our analysis considered live births within 15 years preceding the 2008 NDHS. The risks of death in children below age five were estimated using Cox proportional regression analysis. Results were presented as hazard ratios (HR) with 95% confidence intervals (CI).

**Results.** The study found substantial differentials in under-five mortality by ethnic affiliations. For instance, risks of death were significantly lower for children of the Yoruba tribes (HR: 0.39, CI: 0.37–0.42, *p* < 0.001), children of Igbo tribes (HR: 0.58, CI: 0.55–0.61, *p* < 0.001) and children of the minority ethnic groups (HR: 0.66, CI: 0.64–0.68, *p* < 0.001), compared to children of the Hausa/Fulani/Kanuri tribes. Besides, practices such as plural marriage, having higher-order births and too close births showed statistical significance for increased risks of under-five mortality (*p* < 0.05).

**Conclusion.** The findings of this study stress the need to address the ethnic norms and practices that negatively impact on child health and survival among some ethnic groups in Nigeria.

## Introduction

The relationship between ethnicity and health outcomes has been well established (Wall 1998; Anand 1999; Macbeth and Shetty 2001; Braun 2002; Collins 2004; Culley 2006; Antai 2011a; Powers 2013). This is because different ethnic groups have different sociocultural values and practices that could either positively or negatively impact on health metrics. For instance, purdah system (i.e. wife seclusion) is a common cultural practice among the Hausa tribe in Northern Nigeria and this practice has been cited as a major challenge restricting women's access to health care (Wall 1998). Also emphasizing the influence of cultural practices on health outcomes, Griffiths et al. (2004) established that homogenous nutritional outcomes are found among the children in the same community mainly because of cultural norms regarding food intake. Collins (2004) opined that health disparities will have more to do with differences in sociocultural practices than with genetics.

Specifically, Nigeria is a multi-ethnic country with more than 250 identifiable ethnic groups (NPC and ICF Macro 2009). Besides, the country has a diverse geographical and sociopolitical landscape and is home to many cultural and linguistic groups. Nigeria is by far the most populous country in Africa. According to 2006 population and housing census, the country's population stood at 140,431,790 (National Population Commission 2009). The country's population is estimated to be growing at 3.2% per annum (NPC and ICF Macro 2009). Thus, from the arid northern region of the country to the savannah west, there are varied health outcomes among various population groups – children, youth, adult and the old.

Meanwhile, in spite of the significant improvements in children's health outcomes in the last century, under-five mortality remains unacceptably high in Nigeria. The country's under-five mortality rate of 157 per 1000 live births is among the highest in the world (NPC and ICF Macro 2009). Besides, there are huge regional variations in under-five mortality rate in the country, with a region having as high as 222 deaths per 1000 live births while the rate is 89 per 1000 live births in another region within the country (NPC and ICF Macro 2009). While the former scenario is found among the Hausa/Fulani/Kanuri tribes in the northern region of the country, the latter scenario is reminiscent of the Yoruba ethnic group in the south-west. Worse still, increased risk of child deaths is recently reported in some regions in Nigeria (Adekanmbi, Kayode, and Uthman 2011; Antai 2011a; Aremu et al. 2011).

In effect, addressing poor child health outcomes requires scientific evidence on such important characteristics like ethnic values and norms. Public health and demographic literature show that knowledge about the determinants of child mortality at the individual level is insufficient to address the problem (Antai et al. 2009; Babalola and Fatusi 2009; Becher 2010; Griffiths et al. 2004; Harttgen and Misselhorn 2006; Olalekan and Kongnyuy 2008; Omariba and Boyle 2007; Sastry 1996; Shabani et al. 2010; Boco 2010). This is because factors such as the characteristics of the ethnic group or community where a child is raised tend to affect child's survival chances. It is thus important studying the influence of ethnic values and practices on childhood mortality in such a culturally and religiously diverse setting like Nigeria. Hence, this study examines the influence of ethnic values and practices on under-five mortality in Nigeria.

## Literature review

A realization of the problems associated with the model based on classifying the human population by race when conducting a research identifying factors related to health and disease led to the emergence of ethnicity-based classification schema. This is considered to be complex in that it adds the cultural component which was missing in the race-based one which focused on social and biological components (Anand 1999). Anand (1999) argues that the use of the ethnicity-based classification schema in determining risk factors differentials can have a great impact on public health.

Collins (2004) acknowledged the significance of the ethnicity-based classification schema by showing the link between ethnicity and disease risk or health outcomes through genetic or non-genetic (social, cultural, education and economic) surrogate relationships or a union of the two. On the other hand, Culley (2006, 149) contended that ‘ethnicity is contextual.’ This is because characteristics of the contexts tend to influence health outcomes. Culley (2006) further noted that the link between ethnicity and health outcomes or disease risk is modified by factors such as age, gender and socio-economic status. Additionally, he asserted that the significance of the above factors is determined by context. In other words, it can be said that in as much as ethnic identity is considered to be an important factor determining health outcome, it should not be considered independent of other factors.

Emerging studies have reported on the relationship between ethnicity and child survival (Antai et al. 2010; Omariba and Boyle 2007; Fayeun and Omololu 2011). For instance, examining the determinants of infant and child mortality in Kenya, Omariba, Beaujot, and Rajulton (2007) found that infant mortality was highest among Luo tribe while it was lowest among the Kikuyus. Though child mortality was also highest and lowest among Luo and Kikuyu ethnic groups, respectively, the figure for child mortality was twice higher than that for infant mortality. In addition, the study showed that the variations among the other ethnic groups were larger in child mortality compared to infant mortality.

Moreover, Fayeun and Omololu (2011) found that under-five mortality varied significantly by ethnicity in Nigeria. Antai and Moradi (2010) also established that the link between ethnicity and under-five mortality could be modified by other factors such as maternal education and age. Factors such as maternal education and other socio-economic characteristics have been reported to have an effect on the mother's health-seeking behaviour, such as hospital delivery and child immunization (Antai 2010; Aremu et al. 2011). In effect, levels of educational attainments and attitude towards health seeking tend to vary from one ethnic group to the other.

Other similar studies have found a significant relationship between ethnic affiliation and health outcomes and health care service utilization (Haan et al. 2012; Målqvist et al. 2013; Yen et al. 2013). For instance, Haan et al. (2012) established ethnic differentials in utilization of youth mental health care among selected children of Dutch, Turkish and Moroccan origin. Målqvist et al. (2013) observed ethnic differentials in utilization of health care services in Vietnam. Berg et al. (2012) found that, though the incidence of depressive symptoms seems to be high among smokers, the relationship between smoking and poor health outcomes differs by ethnic affiliation. Powers (2013) found that infant mortality was associated with racial or ethnic affiliation of mothers.

Although, Culley (2006) argues that ethnicity is contextual, ethnicity cannot be regarded as positive or negative. Sastry (1997) contends that child mortality risks could be exacerbated or mitigated depending on the context the children find themselves. For instance, ethnic groups that have developed good health-seeking behaviours are more likely to have better child health outcomes than the group of people that still have poor attitude towards health seeking.

Understanding the ethnic values and practices inimical to child survival can be very helpful in reducing under-five mortality in an ethnically diverse setting like Nigeria. Few studies have examined the association between ethnicity and under-five mortality in Nigeria. But, similar studies on the influences of ethnic values and practices on under-five mortality in Nigeria have been minimal or non-existent. Thus, this study which aims to examine the influences of ethnic values and practices on under-five mortality in Nigeria was designed to fill this gap.

## Data source and methods

### Data source

The data employed in this study were cross-sectional data from the 2008 Nigeria Demographic and Health Survey (NDHS). The survey elicited information on demographic and health indicators at the national, regional and state levels. The primary sampling unit which was regarded as a cluster for the 2008 NDHS is defined on the basis of enumeration areas. Sample for the survey was selected using stratified two-stage cluster design consisting of 888 clusters (NPC and ICF Macro 2009). In all, a nationally representative sample of 36,800 households was selected for the survey. Data were gathered from 33,385 women aged 15–49 and 15,486 men aged 15–59. A detailed report of the data collection methods and procedures for 2008 NDHS has been published elsewhere (NPC and ICF Macro 2009). The present analysis covers the birth history of 33,385 women who had a total of 104,808 live births within the 15 years preceding the 2008 survey (i.e. 1993–2008).

### Ethical consideration

This study utilized a secondary data-set with all identifier information removed. Hence, confidentiality and anonymity are guaranteed. The approval for the conduct of the survey was given by both the Ethics Committee of the Opinion Research Corporation Macro International (USA) and the National Ethics Committee in the Federal Ministry of Health, Nigeria (NPC and ICF Macro 2009). This paper was extracted from a larger study for which permission has been given by ICF Macro to utilize NDHS data.

### Variables measurements

#### Outcome variable

The dependent variable in this study is the risk of under-five mortality – defined as the risk of a live-born child dying between birth and the fifth birthday and this was measured as the duration of survival since birth in months. Our analysis was child-based. That is, birth recode of 2008 NDHS data was utilized and analysis covered the live births born to respondents within 1993–2008 period. The children's survival status and age at death in months (if the child had died) or the last month that they were known to be alive (if child was still living) were combined to generate the outcome variable for the survival analysis. Children known to have died before reaching age five (i.e. non-censored) were considered as cases while children who were still alive at the time of the survey were regarded as right-censored observations in our analysis.

#### Explanatory variables

The choice of explanatory variables for this study was guided by the literature. In particular, Wall's (1998) work on the social contest of maternal morbidity and mortality among the Hausa of Northern Nigeria was helpful and guided us in the choice of relevant and important ethnic values and practices which could influence child survival in Nigeria. These selected explanatory variables include: (1) parity (having many children remains a norm in many parts of Nigeria – parity was measured by number of living children); (2) birth order (having higher-order births is a norm among many ethnic groups in Nigeria); (3) early motherhood (forced sexual cohabitation at puberty and early marriage among Hausa/Fulani/Kanuri are always followed by teenage pregnancy and early motherhood – early motherhood was measured by age at first birth); (4) too close births (short birth intervals often result into too close births – this was measured by preceding birth interval); (5) home delivery (is also a common practice among some ethnic groups in Nigeria – this was measured by ‘place of delivery’); and (6) norm regarding polygynous marriage [plural marriage is part of the cultural practice among many ethnic groups in Nigeria as in other West African countries (Omariba and Boyle 2007) – this norm was measured by ‘type of marriage’]. In addition, because studies have shown that education plays a key role in influencing children's health outcomes (Buor 2003; Kravdal 2004), education was considered in this paper as a control variable – this was measured by highest level of education attained.

In addition, apart from ethnicity covariate – which is the key explanatory variable, we selected other important independent variables to show the profiles of the respondents. These include region of residence, place of residence, religion, wealth index, employment status, maternal age, child's sex and selected partners’ characteristics. Out of the over 250 identifiable ethnic groups in Nigeria, there are three major ethnic groups – Hausa/Fulani/Kanuri, Igbo and Yoruba. The Hausa/Fulani/Kanuri were categorized as one group in this study because the three tribes have common language; common antecedent and common set of customs and values (Antai 2010). All other minority ethnic groups were grouped as one and they were regarded as ‘others’ in this study.

### Statistical analysis

Three levels of analysis (univariate, bivariate and multivariate) were employed in this study. At the univariate level, percentage distribution of respondents was presented. At the bivariate level, Pearson chi-square test was employed to examine relationship between variables. At the multivariate level, data were analyzed using Cox proportional hazard model to explore relationship between under-five mortality and selected explanatory variables. The Cox regression procedure is a useful technique for the analysis of survival data and it takes care of censoring problem in mortality data as some children are not fully exposed to the mortality risk. In social science research, censoring occurs when the value of an observation is not fully known. Cox regression analysis allows for the inclusion of censored data and it models censored time-until-event data as a dependent variable where it can be assumed that the covariates have a multiplying effect on hazard rates. We treated under-five mortality as the time between birth and death of a child under-age five or until the observation is censored.

Thus, the probability of under-five death is called the hazard. The hazard is modelled using the following equation:

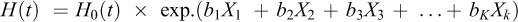
(1)

where *X*_1_ … *X*_*k*_ are a collection of explanatory variables and *H*_0_(*t*) is the baseline hazard at time *t*, representing the hazard for a person with the value 0 for all the explanatory variables. By dividing both sides of Equation (1) by *H*_0_(*t*) and taking logarithms, the Equation (1) becomes:

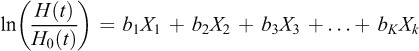
(2)

where *H*(*t*)/*H*_0_(*t*) is regarded as the hazard ratio (HR). The coefficients *b*_*i*_ … *b*_*k*_ are estimated by Cox regression (Cox 1972; Fox 2002).

At the multivariate level, five models were fitted in all. Model 1 presents the univariate HR showing the relationship between under-five mortality and ethnic affiliation. To avoid incorporating highly correlated variables into the same model, Model 2 considered all the variables earlier described as norms and ethnic practices, with the exception of parity; while Model 4 considered all the selected ethnic values and practices with the exception of birth order. Models 3 and 5 adjusted for the effect of parental education. As earlier indicated, education is an important predictor of child survival (Kravdal 2004; Antai 2011a), and it as an important attribute that could loosen men and women from traditional norms and cultural practices which could negatively influence health outcomes. Measures of association between outcome variable and explanatory variables were expressed as HR with 95% level of confidence intervals (CI). All analysis was done using Stata software (version 11.1).

## Results

### Socio-economic and demographic characteristics of study population by ethnic affiliations

The distribution of the study population among the various ethnic groups is presented in Table 1. With the exception of child's sex, all characteristics vary significantly by ethnic affiliations. The largest ethnic group was the Hausa/Fulani/Kanuri tribes (40.3%), followed by the minority ethnic groups (38.6%). Also, Igbo and Yoruba ethnic groups accounted for 11% each. With respect to maternal age, the largest groups were children of mothers aged 35 or older (57.9%). At the country level, more than half of the children (52.1%) were children of mothers with no formal education. A careful examination of maternal education indicates a huge variation by ethnic affiliation. For instance, 85.4% of the children of Hausa/Fulani/Kanuri tribes were children of mothers with no formal education as against only 13.4% among Igbo and 18.7% among Yoruba. Father's education also showed a similar pattern.

**Table 1.** Socio-economic and demographic characteristics of the study sample by ethnicity.

		Ethnic groups				
Variables/categories	Nigeria% (*n*)	Hausa/Fulani/Kanuri % (*n*)	Igbo % (*n*)	Yoruba % (*n*)	Others % (*n*)	*p* Value
Ethnicity	100 (104,808)	40.3 (42,275)	10.6 (11,114)	10.5 (10,971)	38.6 (40,448)	
Child's sex						0.562
Male		51.0	51.2	51.8	51.6	
Female		49.0	48.8	48.4	48.4	
Maternal age						0.000
15–24	8.7	11.2	5.0	5.3	8.2	
25–34	33.4	34.4	30.6	34.0	33.2	
35+	57.9	54.4	64.4	60.6	58.6	
Mother's education						0.000
None	52.1	85.4	13.4	18.7	38.9	
Primary	24.5	10.3	37.5	32.0	33.9	
Secondary	18.6	3.5	38.6	38.3	22.1	
Higher	4.8	0.8	10.4	11.0	5.1	
Father's education						0.000
None	44.4	75.1	12.8	14.9	30.2	
Primary	23.3	11.4	47.4	28.3	27.1	
Secondary/higher	32.3	13.5	39.8	56.8	42.7	
Mother's employment status						0.000
Not working	28.8	44.2	16.3	7.7	22.9	
Working	71.2	55.8	83.7	92.3	77.1	
Father's employment status						0.000
Not working	24.7	37.4	15.0	6.6	19.5	
Working	75.4	62.6	85.0	93.4	80.5	
Wealth index						0.000
Poorest	24.1	38.1	5.9	3.2	21.8	
Poorer	23.3	30.8	11.1	12.1	23.0	
Middle	20.1	16.0	24.5	16.7	24.7	
Richer	17.4	10.1	29.3	26.3	18.4	
Richest	15.1	4.9	29.3	41.7	12.1	
Religious affiliation						0.000
Catholic/other Christianity	43.1	0.6	95.3	54.0	70.8	
Islam	55.1	98.7	0.6	44.7	26.8	
Others	1.8	0.7	4.1	1.3	2.4	
Place of residence						0.000
Urban	28.4	21.0	46.2	52.9	21.6	
Rural	71.6	79.0	53.8	47.1	78.4	
Region						0.000
North-central	13.9	4.1	2.3	10.1	31.1	
North-east	16.5	23.8	0.2	0.3	19.5	
North-west	31.7	70.5	0.5	0.5	8.5	
South-east	9.7	0.1	78.8	0.1	0.6	
South-south	15.1	1.4	9.1	1.2	34.5	
South-west	13.1	0.1	9.1	87.8	5.8	

Hausa/Fulani/Kanuri tribes were found to be mostly unemployed. In addition, children of Hausa/Fulani/Kanuri tribes were most commonly found in the poorest households (38.1%), while 5.9% of Igbo children and 3.2% of Yoruba children were from poorest households. While Igbo mothers were mostly Christians, Hausa/Fulani/Kanuri mothers were predominantly Muslim. Considering the place of residence, the Yoruba-speaking areas were the most urbanized (52.9%) followed by the Igbo-speaking areas (46.2%), while only one in five of the children of Hausa/Fulani/Kanuri tribes were found in the urban centres. Also, while the Igbo ethnic group was predominantly found in the south-east (78.8%), Yoruba in the south-west (87.8%) and the minority ethnic groups in the north-central (31.1%) and south-south (34.5%), the Hausa/Fulani/Kanuri tribes spread over the north-west (70.5%) and the north-east (23.8%).

### Ethnic values and practices by ethnic affiliations

Table 2 presents the percentage distribution of the study population by selected ethnic values and practices according to ethnic affiliations. The results showed that almost two-thirds of the children of Hausa/Fulani/Kanuri tribes (60.4%) were children of mothers whose age at first birth was less than 18 years. In contrast, 27.6% and 23.0% of the children of Igbo and Yoruba tribes, respectively, were children of mothers whose age at first birth was less than 18 years. More than half of the children of the minority ethnic tribes (54.2%), Igbo tribe (53.4%) and Hausa/Fulani/Kanuri tribes (53.2%) were children from households with parity level of 5 or higher, as against 35% among the Yoruba.

**Table 2.** Percentage distribution of study sample by ethnic values and sociocultural practices according to ethnic affiliation.

		Ethnic groups				
Variables/categories	Nigeria% (*n*)	Hausa/Fulani/Kanuri % (*n*)	Igbo % (*n*)	Yoruba % (*n*)	Others % (*n*)	
Mother's age at first birth						0.000
< 18	46.6	60.4	27.6	23.0	46.6	
18+	53.4	39.6	72.4	77.0	53.4	
Parity						0.000
<3	16.7	16.4	15.7	19.4	16.3	
3–4	32.2	30.4	30.9	45.6	29.5	
5+	51.1	53.2	53.4	35.0	54.2	
Family type						0.000
Monogamous	61.3	50.1	85.3	67.0	65.1	
Polygynous	38.7	49.9	14.8	33.0	34.9	
Birth order						0.000
1	23.0	20.4	24.1	28.9	23.2	
2–4	47.8	45.0	49.7	54.9	47.9	
5+	29.2	34.6	26.2	16.2	28.9	
Birth interval						0.000
<24 months	98.1	98.6	98.4	96.7	98.0	
24+	1.9	1.4	1.6	3.3	2.0	
Place of delivery						0.000
Home	63.9	91.4	20.8	22.1	61.6	
Health facility	36.1	8.6	79.2	77.9	38.4	

Considering birth order, more than one-third (34.6%) of the children of Hausa/Fulani/Kanuri tribes were children of birth order 5 or higher as against 16.2% of children of Yoruba tribes. An overwhelmingly high proportion of children of each of the four categories of ethnic groups were born after preceding birth interval of less than 24 months, with the percentage ranging from 96.7% among Yoruba to 98.6% among the Hausa/Fulani/Kanuri tribes. With respect to place of delivery, majority of Hausa/Fulani/Kanuri children (91.4%) and children of the minority ethnic groups (61.6%) were delivered at home as against 20.8% and 22.1% among Igbo and Yoruba tribes, respectively. Table 2 further showed that half of the children of Hausa/Fulani/Kanuri tribes (49.49%), 34.9% of the children of the minority ethnic groups, 33.0% of the children of Yoruba tribe and 14.8% of the children of Igbo tribe were children of mothers in polygynous unions.

### Socio-economic and demographic factors associated with under-five mortality

The bivariate relationship between under-five mortality and ethnic affiliation and other selected background characteristics is presented in Table 3. The results indicate that all characteristics were significantly associated with under-five mortality, with the exception of father's employment status. The relationship between ethnic affiliation and under-five mortality showed that lowest percentage of under-five death was found among the Yoruba tribe (9.2%) and highest among the Hausa/Fulani/Kanuri tribes (23.0%; *p* < 0.001). In agreement with theoretical expectation, results showed that slightly more deaths occurred among male children (17.7%) than among females (16.4%; *p* < 0.001). Considering other characteristics, results revealed that high percentage of under-five death was reported for children of mothers aged 35 or older (18.4%, *p* < 0.001); children of the Muslim mothers (20.7%); children of women in rural areas (19.0%); children of women with no formal education (21.5%); and children of mothers in the poorest wealth quintile (22.5%). Results also revealed that percentage of under-five death was lowest in the south-west (9.1%) and highest in the north-east (22.1%) and north-west (22.8%).

**Table 3.** Background characteristics associated with under-five mortality in Nigeria.

	Under-five (0–59 months)			
Variables/categories	Alive (*n* = 86,687)	Dead (*n* = 18,121)	Total number (*n* = 104,808)	Chi-square
Ethnic origin				191.6***
Hausa/Fulani/Kanuri	77.0	23.0	42,275	
Igbo	87.6	12.4	11,114	
Yoruba	90.8	9.2	10,971	
Others	85.3	14.7	40,448	
Child's sex				26.2***
Male	82.3	17.7	53,773	
Female	83.6	16.4	51,035	
Maternal age				50.5***
15–24	85.4	14.6	9237	
25–34	84.6	15.4	35,084	
35+	81.6	18.4	60,487	
Wealth index				173.6***
Poorest	77.5	22.5	28,544	
Poorer	78.9	21.1	25,704	
Middle	83.6	16.4	21,197	
Richer	87.6	12.4	16,878	
Richest	91.7	8.3	12,485	
Religion affiliation				145.6***
Catholic/other Christianity	87.5	12.5	43,128	
Islam	79.3	20.7	58.526	
Others	83.4	16.6	2297	
Mother's education				241.7***
None	78.5	21.5	58,550	
Primary	85.3	14.7	24,785	
Secondary	89.7	10.3	17,039	
Higher	92.8	7.2	4434	
Father's education				325.6***
None	77.6	22.4	48,788	
Primary	84.6	15.4	22,597	
Secondary or higher	88.9	11.1	31,130	
Mother's employment status				18.4***
Currently working	28.3	31.1	31,462	
Not working	71.7	68.9	73,346	
Father's employment status				3.5
Working	82.2	17.8	27,055	
Not working	83.2	16.8	77,753	
Place of residence				147.8***
rban	87.9	12.1	26,574	
Rural	81.0	19.0	78,234	
Region				114.8***
North-central	86.9	13.1	18,631	
North-east	77.9	22.1	3995	
North-west	77.2	22.8	29,338	
South-east	86.4	13.6	9292	
South-south	87.1	12.9	12,006	
South-west	90.9	9.1	11,546	

*** *p* < 0.001.

### Ethnic values and practices associated with under-five mortality

Table 4 presents the results of bivariate relationship between under-five mortality and the selected ethnic values and practices. The results indicate that all the selected variables were significantly associated with under-five mortality. Considering mother-level characteristics, high proportion of under-five death was found among children of mothers in polygynous unions (20.6%, *p* < 0.001); and among children of women whose first birth occurred earlier than 18 years (20.0%, *p* < 0.001). With respect to the selected child-level variables, under-five death was high among children of birth order five or higher (19.9%); among children delivered at home (12.1%); and among children born after the birth interval of less than two years (17.4%).

**Table 4.** Ethnic values and sociocultural practices associated with under-five mortality in Nigeria.

	Under-five (0–59 months)			
Variables/categories	Alive (*n* = 86,687)	Dead (*n* = 18,121)	Total number (*n* = 104,808)	Chi-square
Mother's age at first birth				129.0*
<18	80.0	20.0	50,091	
18–34	85.5	14.5	54,516	
35+	87.6	12.4	201	
Parity				232.0*
<3	74.9	25.1	17,237	
3–4	82.2	17.8	33,073	
5+	86.0	14.0	54,498	
Family type				169.1*
Monogamous	85.0	15.0	58,081	
Polygynous	79.4	20.6	39,210	
Birth order				93.7*
1	83.7	16.3	23,751	
2–4	84.3	15.7	49,851	
5+	80.1	19.9	31,206	
Birth interval				119.9*
<24 months	82.6	17.4	79,328	
24+	93.9	6.1	1468	
Place of delivery				56.2*
Home	87.8	12.1	18,990	
Health facility	91.5	8.5	8958	

* *p* < 0.001.

### Risk factors of under-five mortality: survival analysis

**Figure 1.**
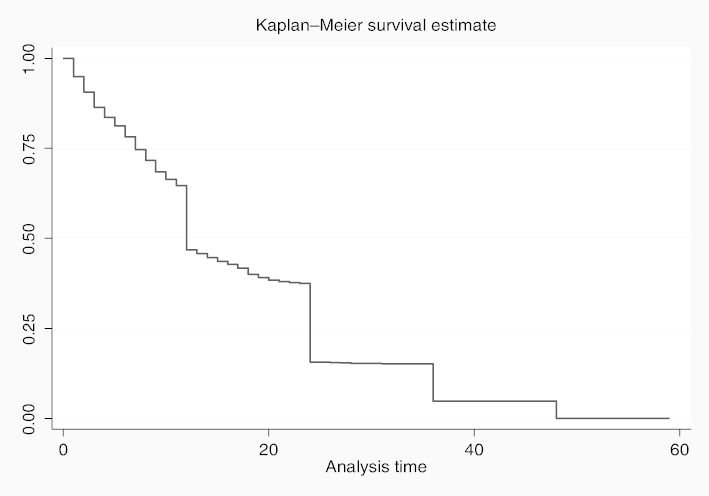
Child survival plot for children that died before reaching age five among all live-born children within 1993–2008 (15 years before the survey).

**Figure 2.**
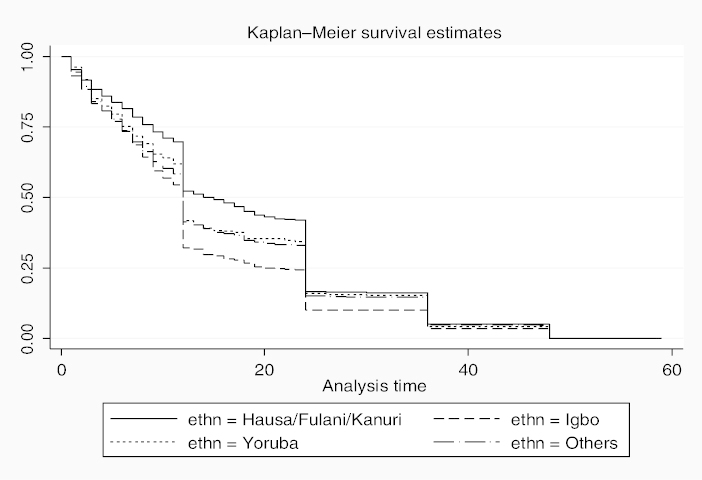
Child survival plot by ethnic affiliation for children that died before fifth birthday among all live-born children within 1993–2008.

The results of survival analysis are presented in Table 5. In all, five models were fitted to examine the risk factors of under-five mortality. Model 1 presents the univariate HR to examine the relationship between ethnic affiliation and under-five mortality. Models 2–5 present the multivariate HR to examine the effects of selected ethnic norms and practices on under-five mortality. Considering that parity and birth order were found to be correlated (analysis not shown), we incorporated each of the two variables into separate models. Also, we adjusted for the effects of maternal and paternal education in Models 3 and 5. For the description of the survival curves and functions, child survival plot (Figure 1) is presented to show the duration of survival since birth for children that died during the first five years of life (0–59 months). Also, further description of the mortality risks among the children by ethnic affiliations is provided by Figure 2.

**Table 5.** Hazard ratios (HR) and 95% confidence intervals (CI) for ethnic and sociocultural factors associated with under-five mortality, Nigeria 2008.

	Model 1	Model 2	Model 3	Model 4	Model 5
Variables	HR (95% CI)	HR (95% CI)	HR (95% CI)	HR (95% CI)	HR (95% CI)
Ethnicity					
Hausa/Fulani/Kanuri	1	1	1	1	1
Igbo	0.58 (0.55–0.61)***	0.91 (0.77–1.07)	1.00 (0.84–1.20)	0.97 (0.82–1.15)	1.12 (0.93–1.34)
Yoruba	0.39 (0.37–0.42)***	0.51 (0.42–.62)***	0.57 (0.47–0.70)***	0.55 (0.45–0.67)***	0.63 (0.52–0.78)***
Others	0.66 (0.64–0.68)***	0.87 (0.80–0.96)**	0.93 (0.84–1.03)	0.90 (0.82–0.99)*	0.98 (0.88–1.08)
Mother's age at first birth					
<18		1	1	1	1
18+		0.94 (0.86–1.02)	0.95 (0.87–1.04)	0.86 (0.79–0.94)**	0.89 (0.81–0.97)**
Birth order					
1		1	1	–	–
2–4		0.92 (0.89–0.96)	0.92 (0.89–0.96)	–	–
5+		1.78 (1.62–1.95)***	1.73 (1.58–1.90)***	–	–
Birth interval					
<24		1	1	1	1
24+		0.54 (0.38–0.78)**	0.50 (0.34–0.73)***	0.55 (0.38–0.79)**	0.50 (0.34–0.74)***
Place of delivery					
Home		1	1	1	1
Health facility		0.92 (0.82–1.03)	1.00 (0.89–1.12)	0.89 (0.79–0.98)*	1.00 (0.89–1.13)
Family structure					
Monogamous		1	1	1	1
Polygynous		1.61 (1.46–1.77)***	1.57 (1.42–1.72)***	1.48 (1.35–1.64)***	1.42 (1.28–1.57)***
Parity					
<3		–	–	1	1
3–4		–	–	0.47 (0.42–0.52)***	0.46 (0.41–0.51)***
5+		–	–	0.45 (0.40–0.51)***	0.43 (0.38–0.49)***
Mothers’ education					
No education		–	1	–	1
Primary		–	0.98 (0.87–1.11)	–	1.02 (0.90–1.15)
Secondary or higher		–	0.79 (0.68–0.93)**	–	0.73 (0.62–0.85)***
Father's education					
No education		–	1	–	1
Primary		–	0.94 (0.83–1.06)	–	0.91 (0.81–1.03)
Secondary or higher		–	0.89 (0.79–1.012)	–	0.85 (0.75–0.96)**

* *p* < 0.05, **p < 0.01, ***p < 0.001.

Results from the univariate hazard model (Model 1; Table 5) indicate a significant relationship between the risk of under-five mortality and ethnic affiliation. For instance, the results showed that risks of death were 61% significantly lower for children of the Yoruba tribes (HR: 0.39, CI: 0.37–0.42, *p* < 0.001), 42% significantly lower for children of Igbo tribes (HR: 0.58, CI: 0.55–0.61, *p* < 0.001) and 34% significantly lower for children of the minority ethnic groups (HR: 0.66, CI: 0.64–0.68, *p* < 0.001), compared to children of the Hausa/Fulani/Kanuri tribes. After incorporating other selected factors into the analysis in Model 2, the results still indicate significantly lower risks of death for children of Yoruba tribe (HR: 0.51, CI: 0.42–0.62, *p* < 0.001) and children of the minority ethnic groups (HR: 0.87, CI: 0.80–0.96, *p* < 0.01) relative to children of the Hausa/Fulani/Kanuri tribes. Model 2 further indicates a significant relationship between under-five mortality and factors such as, family structure, birth order and preceding birth interval. For instance, risks of death were 61% significantly higher for children of mothers in polygynous unions (HR: 1.61, CI: 1.46–1.77, *p* < 0.001) and almost two-folds higher for children of fifth- or higher-order birth (HR: 1.78, CI: 1.62–1.95, *p* < 0.001). In contrast, risks of death were significantly lower for children with longer preceding birth interval (HR: 0.54, CI: 0.38–0.78, *p* < 0.001) relative to children with shorter birth interval.

After adjusting for the effects of parental education in Model 3 (Table 5), the results indicate similar findings. For instance, results indicate a significantly lower risk of death for children of the Yoruba tribe (HR: 0.57, CI: 0.47–0.70, *p* < 0.001) and children of the minority ethnic tribes (HR: 0.87, CI: 0.80–0.96, *p* < 0.01), compared with children of the Hausa/Fulani/Kanuri tribes. Even, the characteristics such as family structure, birth order and birth interval remain significantly associated with under-five mortality. Model 3 further indicates that the risks of death were 21% significantly lower for children whose mothers had at least secondary education (HR: 0.79, CI: 0.68–0.93, *p* < 0.01) relative to those whose mothers had no formal education. Maternal education seems to be more important for child survival than paternal education which was insignificantly associated with under-five mortality.

Model 4 which contains ethnicity covariate and other selected factors (excluding birth order) indicates significantly lower risks of death for children of Yoruba tribes (HR: 0.55, CI: 0.45–0.67, *p* < 0.001) and children of the minority ethnic groups (HR: 0.90, CI: 0.82–0.99, *p* < 0.05), compared with children of the Hausa/Fulani/Kanuri tribes. Model 4 further indicates significant association between child survival and mother's age at birth, place of delivery, family structure, birth interval and parity (*p* < 0.001). Additionally, although inclusion of education covariate in Model 5 (Table 5) leads to a slight change in the estimates, the results in Model 5 are comparable to those in Models 3 and 4.

## Discussion

The objective of this paper was to examine the influences of ethnic values and practices on the observed inequality in under-five mortality in Nigeria. After running Cox regression analyses separately for the three major ethnic groups and also for the minority ethnic groups (analysis not shown), we found results similar to those reported in Table 5.

Our results indicate a significant relationship between under-five mortality and ethnic affiliation, thus supporting Antai's (2011a) study which established significant variation in under-five mortality by ethnicity in Nigeria. Our analysis also revealed significantly higher risks of death for children of higher-order birth and children with a short preceding birth interval. These results have some policy implications. Cultural factors such as the practice of having short intervals between births and having higher-order births have grave implications for child survival. As long as cultures favourable to high fertility and too close births are sustained among some ethnic groups as observed in some sections of the country, regional inequality in under-five mortality will continue to persist in Nigeria.

Further, risks of death were found to be significantly higher for children of mothers in polygynous marriages compared to children of mothers in monogamous unions. Expectedly, children in polygynous families are likely to suffer poorer health outcomes compared to those in monogamous families. Plausible explanation for this is that the presence of many women and many children in the same household could engender a stiff competition for household resources, especially if the household is poor. Besides, polygyny can have great implications for child's health outcomes and survival because of family conflict and discord among children and unhealthy rivalry among co-wives. Omariba and Boyle (2007) had earlier observed that culture of polygyny is more prevalent in West Africa than in other part of the continent. Our results indicate a huge ethnic variation with respect to the practice of polygynous marriage. For instance, the rate of polygyny found among Hausa/Fulani/Kanuri tribes was twice the rate found among the Yoruba. While the culture of polygyny is becoming less fashionable among the Yoruba and Igbo tribes, the practice still persists among the Hausa/Fulani/Kanuri tribes and among the minority ethnic groups. This perhaps has partly accounted for the observed ethnic inequality in under-five mortality in the country.

The Yoruba ethnic group is predominantly found in the south-western part of Nigeria (Table 1). Our analysis indicates that this is the region with the lowest rate of under-five mortality in the country. Results also indicate the Yoruba as highly educated tribe. Besides, high-fertility-related culture is becoming less fashionable among them. For instance, only a small proportion of the Yoruba children were from high-parity households. Also, the culture of postponement of marriage and childbearing has been well ingrained among the Yoruba, as only a small proportion of the women had given births before the age of 18. Further, majority of the children of Yoruba tribe were born after the preceding birth interval of two or more years and only small proportion belonged to birth order 5 or higher. In addition, the culture of seeking medical attention and having hospital delivery seemed well entrenched among the Yoruba, as majority of the children were delivered at a health facility.

In contrast, the Hausa/Fulani/Kanuri tribes are the tribes with the highest proportion of under-five mortality in Nigeria (Table 3). These tribes predominantly live in the north-east and north-western region of the country (Table 1) – the regions with the highest rates of under-five mortality in the country. Apart from the multivariate results discussed earlier, bivariate results indicate Hausa/Fulani/Kanuri tribes as mostly uneducated, mostly unemployed, mostly found in the poorest wealth quintile, relatively young mothers at the birth of the first child and mostly found in the rural areas (Table 1). As earlier reported (Wall 1998), our results showed that Hausa/Fulani/Kanuri tribes were mostly practising cultural practices such as early motherhood (many had first birth earlier than 18 years), too many births (high parity) and too close births (high proportion of the children had preceding birth interval of less than 24 months; Table 3). Implication of factors such as short birth intervals for under-five mortality has been reported in earlier studies in Nigeria (Antai 2011a) and elsewhere (Omariba, Beaujot, and Rajulton 2007). In addition, culture of having many children still persists among the Hausa/Fulani/Kanuri tribes as many children were of higher-order births. Besides, polygynous family structure is still a predominant practice among the Hausa/Fulani/Kanuri tribes as a high proportion of the children were from polygynous family (Table 2).

There are substantial variations in under-five mortality, particularly between Hausa/Fulani/Kanuri tribes and the Yoruba tribe. These variations could be attributable to the differences in ethnic values and cultural practices between these tribes as established in this study. Findings of this study suggest that while the practices that are inimical to child health and survival (such as early childbearing, having too close births and poor health seeking) are becoming less fashionable among the Yoruba tribe, the Hausa/Fulani/Kanuri tribes appear to hold tenaciously to these cultural practices.

Differences in level of educational attainment between Hausa/Fulani/Kanuri tribes and the Yoruba tribe also seem to have brought about variations in under-five mortality between these tribes. Further, huge differentials observed in under-five mortality between Hausa/Fulani/Kanuri and Yoruba tribes could be attributable not only to variables included in our analyses, but also to other variables not considered in these analyses. As earlier observed by Adedini et al. (2012), Adekanmbi, Kayode, and Uthman (2011) and Antai (2011b), other factors such as living conditions within the communities, availability or non-availability of health facility, distance to health facility, differences in levels of regional development and varying economic resources and opportunities, among other factors, may have contributed to the substantial variations in under-five mortality between the Hausa/Fulani/Kanuri and Yoruba tribes.

Our study established that the regional inequalities in under-five mortality in Nigeria are mostly driven by the childhood mortality levels among Hausa/Fulani/Kanuri – the tribes that still mostly practise the culture of early motherhood, high fertility, too close births and worse still are mostly uneducated. The study suggests that, although, the Igbo tribe and the minority ethnic groups still practise some of these risky practices, results suggest that these practices are already becoming less fashionable among them. This may not be unconnected to the effect of education. While the proportion of educated people was high among Igbo, Yoruba and minority ethnic groups, a lot still needs to be done to ensure the attainment of Millennium Development Goal 2 (achieve universal primary education) among the Hausa/Fulani/Kanuri tribes. Also, to ensure the attainment of Millennium Development Goal 4 (reduction in under-five mortality rate) in Nigeria, there is need to address regional inequalities in under-five mortality in the country. In order to address the high regional inequalities in under-five mortality in Nigeria, strategies are needed to address such ethnic values and practices that negatively impact on child health and survival among various ethnic groups in the country. The inequalities in under-five mortality in the country could be substantially reduced if the widely held norms and cultural practices inimical to child health and survival are dropped.

## Limitation

This study is not without its limitations. First, being an analysis of a secondary data-set, the major limitation of this study is the non-inclusion of other important sociocultural factors in our analyses due to the absence of such relevant information in the NDHS data-set. Some of these sociocultural factors are feeding practice, wife seclusion (which could hinder appropriate health seeking), female genital cutting and male circumcision. Further studies on the cultural factors influencing regional variations in under-five mortality are needed in Nigeria. Second, while interpreting the results of this study, it should be borne in mind that this study was based on self-reported information from women. As a result, there is likelihood of reporting bias. In addition, reporting bias may likely vary across the various ethnic groups and this might influence the findings of this study. Third, being a cross-sectional data, a cause–effect relationship could not be established in this study.

## Key messages

What is known on this topic:

1. Few studies have examined the association between ethnicity and under-five mortality in Nigeria.
2. Substantial regional inequalities in under-five mortality exist in Nigeria, with the rate ranging from 89 deaths per 1000 live births in the south-west to 222 deaths per 1000 live births in the north-east region.

What this study adds:

1. The high under-five mortality rate in Nigeria is significantly driven by the childhood mortality levels among the Hausa/Fulani/Kanuri tribes.
2. Some ethnic values and cultural practices significantly explain regional inequalities in under-five mortality in Nigeria.
3. While childhood mortality rates may continue to decline in some regions in the country, the rates will continue to increase among some tribes in some other regions if their culture of early childbearing, polygyny and high-fertility-related norms persist.
